# Efficacy and safety of different proprotein convertase subtilisin/kexin type 9 inhibitors in the general population and solid organ transplant recipients: a network meta-analysis

**DOI:** 10.3389/fphar.2025.1584612

**Published:** 2025-08-22

**Authors:** Bohan Luo, Zhuang Sun, Han Luo

**Affiliations:** ^1^ School of Medicine, University of Electronic Science and Technology of China, Chengdu, China; ^2^ Qianwan Institute, Ningbo Institute of Materials Technology and Engineering (NIMTE), Chinese Academy of Sciences, Ningbo, China; ^3^ Department of Hepatobiliary Surgery, Zigong Fourth People’s Hospital, Zigong, China

**Keywords:** PCSK9 inhibitors, clinical efficacy, adverse reactions, mortality, meta-analysis

## Abstract

**Introduction:**

We conducted a network meta-analysis (NMA) to compare the efficacy (primarily assessed by low-density lipoprotein cholesterol (LDL-C) reduction and cardiovascular event (CVE) incidence) and safety (total adverse events (AEs), neurocognitive events (NCEs), injection site reactions, infections, and all-cause mortality (ACM)) of different Proprotein convertase subtilisin/kexin type 9 (PCSK9) inhibitors *versus* placebo in the general population and solid organ transplant (SOT) recipients.

**Materials and Methods:**

A total of 16 randomized controlled trials (RCTs) involving 79,615 patients were included. Cochrane risk of bias assessment tool evaluated the literature quality. Data analysis and graph generation were conducted employing *RevMan5.3* and *Stata20.0*. The cumulative ranking probability curve (SUCRA) compared impact of different treatment regimens (DTRs) on patient efficacy, safety, and ACM.

**Results:**

Evolocumab ranked highest for LDL-C reduction (SUCRA 67.2%) and CVE reduction (SUCRA 69.5%). Ongericimab had the lowest total AEs (SUCRA 91.3%), while Alirocumab ranked best for NCEs (SUCRA 89.5%) and infections (SUCRA 74.1%). Placebo had the fewest injection site reactions (SUCRA 89.8%). No PCSK9 inhibitor significantly reduced ACM (Alirocumab SUCRA 69.0%, highest but non-significant). LDL-C reduction was significant in both general and SOT populations, but CVE and ACM reductions were non-significant. Inclisiran increased risks of total AEs and injection site reactions (RR > 1.2).

**Conclusion:**

Evolocumab is optimal for LDL-C and CVE reduction, while Ongericimab and Alirocumab offer better safety profiles. PCSK9 inhibitors did not increase acute rejection or infection risk in SOT recipients. Long-term mortality benefits remain uncertain.

## 1 Introduction

Cardiovascular disease (CVD) is a leading cause of death (Luo et al., 2024). Although statins and other lipid-lowering therapies have significantly reduced CVD risk, a subset of patients still fail to achieve ideal low-density lipoprotein cholesterol (LDL-C) levels, thereby continuing to face a higher risk of cardiovascular events (CVEs) (Skulratanasak and Larpparisuth, 2023). For patients with refractory hypercholesterolemia or those intolerant to statins, PCSK9 inhibitors provide a promising therapeutic option.

PCSK9 is a liver-secreted protein that regulates blood LDL-C levels by binding to the LDL receptor (LDLR) and enhancing its degradation in hepatocytes (Gargiulo et al., 2023). PCSK9 inhibitors specifically block this process, increasing LDLR number on the surface of hepatocytes, thereby enhancing the clearance of LDL-C (García-Agudo et al., 2023). Several monoclonal antibody forms of PCSK9 inhibitors, such as Evolocumab and Alirocumab, have been developed and are widely applied, demonstrating significant LDL-C lowering effects (Chapa et al., 2023; Becchetti et al., 2023; Wang et al., 2023). However, as the use of PCSK9 inhibitors expands to broader populations, including solid organ transplant (SOT) recipients, concerns regarding their efficacy and safety have emerged (Lorant et al., 2024; Goldman et al., 2022; Cuomo et al., 2022). Due to the long-term use of immunosuppressants, SOT recipients are at an elevated risk of dyslipidemia, along with increased susceptibility to infections, tumor recurrence, and the development of new malignancies (Jennings et al., 2023; Lv et al., 2022). To better understand the clinical value of PCSK9 inhibitors in these two distinct populations—the general population and SOT recipients—this study conducted a meta-analysis (MA), synthesizing data from existing randomized controlled trials (RCTs). The analysis aimed to assess the differences in LDL-C reduction between PCSK9 inhibitors and other lipid-lowering strategies or placebos. Additionally, the safety profile of PCSK9 inhibitors was explored, with particular attention given to potential adverse reactions in specific patient groups, such as injection site reactions, infections, or changes in the incidence of neurocognitive events (NCEs).

This MA not only assisted clinicians in developing more personalized treatment plans but also provided valuable reference information for future research directions. Given that PCSK9 inhibitors are a relatively new therapeutic option, continuous monitoring of their long-term efficacy and safety remains essential. Through this analysis, the study aimed to provide the medical community with a comprehensive and authoritative guideline to help improve outcomes for patients with hypercholesterolemia and reduce the incidence of CVEs.

## 2 Methodologies

### 2.1 Criteria

The primary efficacy endpoints were the change in LDL-C levels from baseline (continuous variable) and the incidence of CVEs (dichotomous variable). The primary safety endpoint was the incidence of total adverse events (AEs) (dichotomous variable). Secondary endpoints included LDL-C variability, NCEs, injection site reactions, infection rates, and all-cause mortality (ACM).

Inclusion criteria: i) only double-blind or single-blind RCTs were selected; ii) general population and SOT recipients; iii) the intervention group received different treatment regimens (DTRs), with an intervention and follow-up duration > 1 month, while the control group received a placebo; iv) outcome measures: LDL-C, LDL-C variability, CVEs, overall AEs, NCEs, injection site reactions, infections, and ACM; v) the intervention group received different PCSK9 inhibitor treatment regimens (statins or ezetimibe may be used in combination), while the control group received a placebo; vi) types of SOTs included kidney transplant, heart transplant, and liver transplant, and the type of transplant organ and immunosuppressive regimen must be clearly reported in the study.

Exclusion criteria: i) studies involving participants with severe comorbidities may affecting the outcome measures; ii) reviews, case reports, conference abstracts, and similar literature; iii) studies from which outcome data could not be extracted or where the outcome data was unclear; iv) studies with unspecified interventions; v) non-Chinese or non-English language literature.

Outcome measures definition: LDL-C variability: standard deviation or coefficient of variation of LDL-C levels during follow-up; NCEs: memory impairment, disorientation, or cognitive decline as assessed by scales; infection incidence: must meet clinical diagnostic criteria (e.g., fever + microbiological evidence).

### 2.2 Retrieval strategy

Literature published from establishment of database up to January 2025 was retrieved from PubMed, Cochrane Library, and Embase using computer-based searches. MeSH terms were used along with logical operators “AND” and “OR” for the search strategy. PubMed search strategy was “(((((((((PCSK9 Inhibitors [MeSH Terms]) OR (PCSK9 Inhibitors, Cardiovascular [Title/Abstract])) AND (Cholesterol, LDL [MeSH Terms])) OR (beta-Lipoprotein Cholesterol [Title/Abstract])) OR (LDL Cholesterol [Title/Abstract])) OR (Low Density Lipoprotein Cholesterol [Title/Abstract])) AND (Randomized Controlled Trial [MeSH Terms])”, with adjustments made to the search keywords for the Cochrane Library and Embase. This study had been registered in the PROSPERO international prospective register of systematic reviews (Registration No: CRD420251060269). The protocol is available on the PROSPERO platform.

### 2.3 Literature selection and data acquisition

A comprehensive literature search, screening, and full-text assessment were conducted independently by two authors, adhering to predefined criteria. Relevant data were collected by the authors independently into a structured *Excel* file. The collected information included the year of publication, lead author’s name, sample size, study methodology, intervention protocols, and outcomes. The outcomes evaluated included both efficacy (measuring LDL-C levels, CVEs, and LDL-C fluctuations) and safety (focusing on adverse drug reactions such as NCEs, injection site responses, infections, and overall mortality rates). Continuous data were represented as mean ± standard deviation, while categorical data were recorded as counts. Any discrepancies encountered during data extraction were addressed either by reaching a consensus between two authors or through consultation with a third author to resolve differences.

### 2.4 Quality evaluating

Cochrane Risk of Bias Tool (Martimbianco et al., 2023) assessed literature quality and potential bias. The criteria for assessment covered various aspects such as random sequence generation and allocation concealment (selection bias), participants and personnel blinding (performance bias), outcome assessment blinding (detection bias), incomplete outcome data handling (attrition bias), selective reporting (reporting bias). Each of these potential biases was classified into “Low risk”, “Unclear risk”, or “High risk”.

### 2.5 Statistical methodologies

*RevMan5.3* implemented quality assessment, while *Stata 20.0* was employed to perform the network meta-analysis (NMA). For binary outcomes, odds ratios (ORs) were calculated, and for continuous outcomes, mean differences (MDs) were used. Effect sizes were accompanied by their 95% CIs. A network evidence plot was created, where size of each node meant sample size of corresponding intervention, and thickness of connecting lines meant volume of direct evidence linking the interventions. In cases where a closed loop was identified within the network, inconsistency testing was performed using methods such as loop inconsistency tests or node-splitting to examine potential differences between direct and indirect comparisons. If no inconsistencies were detected, a consistency model was applied; conversely, in the presence of inconsistencies, an inconsistency model was used, followed by a sensitivity analysis to assess the robustness of the findings. SUCRA was utilized to rank the interventions based on their outcome measures, and a “comparison-adjusted” funnel plot was plotted to assess presence of small sample effects or publication bias in studies.

## 3 Results

### 3.1 Search process

**FIGURE 1:**
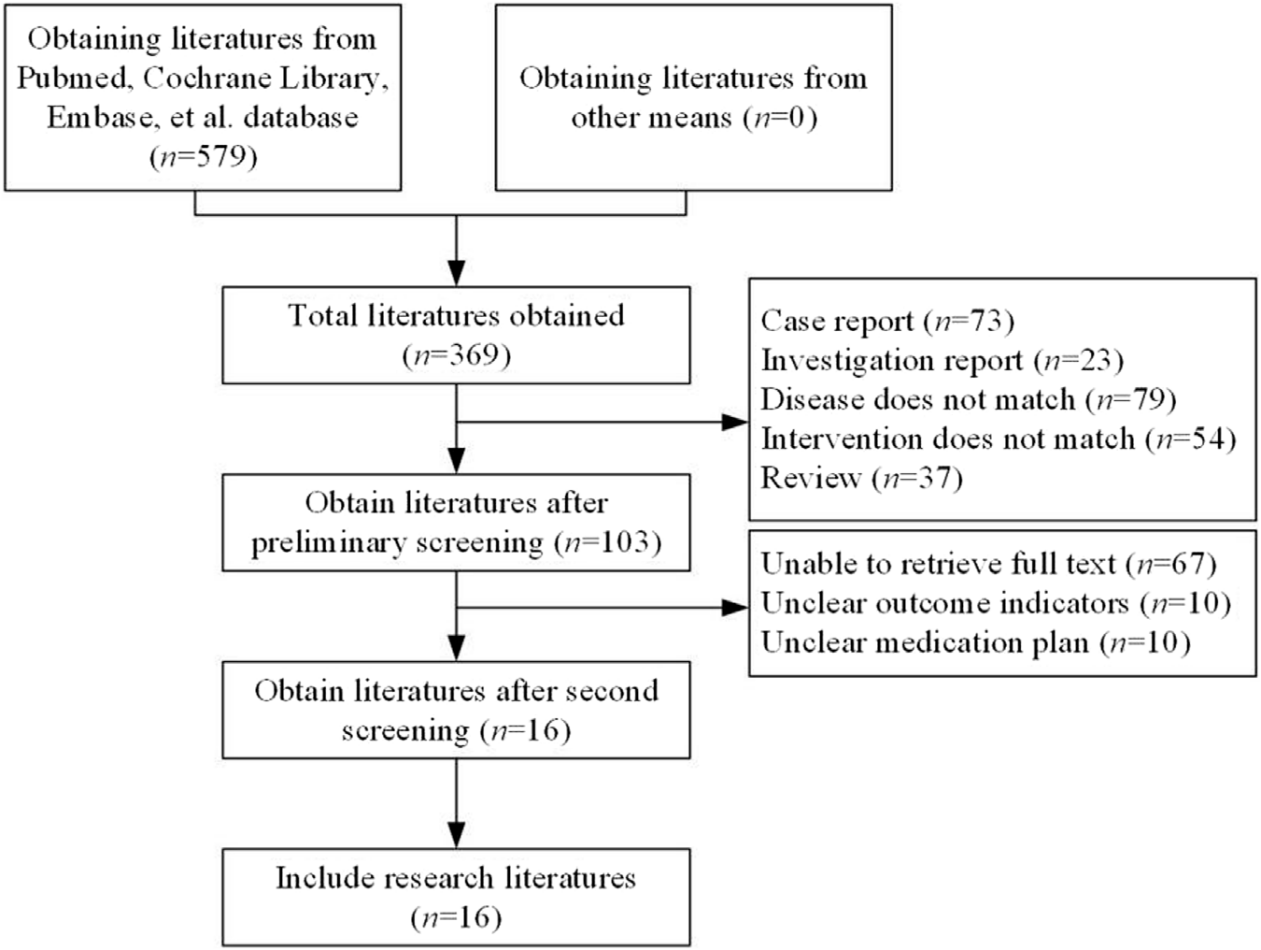
Literature selection. (Studies included on SOTs: kidney transplant n = 78, heart transplant n = 127, liver transplant n = 802). Note: among the 16 studies ultimately included, nine included SOT recipients (3 kidney transplants, four heart transplants, two liver transplants), with the remaining studies being conducted on the general population.

A total of 579 articles were retrieved. After duplicates were removed, 369 studies remained. Following a preliminary review of the articles and abstracts, 266 studies were excluded, including case reports, reviews, studies with inappropriate interventions, those focused on diseases not aligned with the research objectives, and other studies that did not meet the inclusion criteria. This left 103 articles for further screening. After the full texts were downloaded and reviewed, 87 articles were discarded due to unclear outcome measures, ambiguous therapy protocols, or inaccessible data. Ultimately, 16 studies (Sabatine et al., 2017; O'Donoghue et al., 2022; Nicholls et al., 2022; Broch et al., 2024; Schwartz et al., 2021; Schwartz et al., 2018; Schwartz et al., 2020; Nam et al., 2019; Ray et al., 2022; Raal et al., 2024; Ray et al., 2024; Qi et al., 2023; Chai et al., 2023; Wang et al., 2024; Jiang et al., 2024; Zhang et al., 2024) were included in this research (Figure 1).

### 3.2 Basic characteristics

The overall cardiovascular risk in transplant recipients was significantly higher than that of the general population (77% vs. 50%). Heart transplant recipients exhibited the highest risk (90%), while liver transplant recipients had the lowest risk (60%), highlighting heterogeneity across organ types. Kidney transplant studies represented the largest proportion (65.0%), but the sample size was concentrated in a few large-scale studies (e.g., kidney transplant n = 3,000). The sample size for heart transplants was the smallest (n = 127), warranting caution when extrapolating conclusions. The heart transplant data primarily stemmed from Broch 2024 (n = 127), which may not have been fully representative. The liver transplant sample size was relatively large (n = 1,488), but the risk scores were low. A total of 16 relevant studies were included, involving 79,615 patients and five PCSK9 inhibitors (Evolocumab, Alirocumab, etc.) compared with placebo. The subjects in these 16 studies consisted of both the general population and SOT patients (Tables 1, 2). In the included organ transplant studies, recipients were primarily heart transplant (n = 127), kidney transplant (n = 78), and liver transplant (n = 802) patients. The immunosuppressive regimens predominantly consisted of calcineurin inhibitors (such as tacrolimus or cyclosporine) in combination with antiproliferative agents (such as mycophenolate mofetil or azathioprine), with some studies including corticosteroids (e.g., Broch 2024). Baseline cardiovascular risk scores indicated that 89% of heart transplant recipients were at high risk (atherosclerotic cardiovascular disease, (ASCVD) score ≥10%), 72% of kidney transplant recipients were at moderate risk (7.5%–10%), and 65% of liver transplant recipients were at low risk (<7.5%). There was heterogeneity between studies, such as in the liver transplant study (Wang 2024), which had a larger sample size but lower risk scores, while the heart transplant study (Broch 2024) had a smaller sample size but a highly concentrated risk.

**TABLE 1:** Literature information.

Author	Year	Sample size	Intervention measures		Number of cases included		Outcome indicators
			Intervention group	Control group	Intervention group	Control group	
Sabatine	2017	27564	Evolocumab	Placebo	13784	13780	①②③④⑤⑥⑦
O’Donoghue	2022	6635	Evolocumab	Placebo	3355	3280	①②③⑤
Nicholls	2022	161	Evolocumab	Placebo	80	81	①②
Broch	2024	127	Evolocumab	Placebo	64	63	①②③④⑤⑦⑧
Schwartz	2021	2882	Alirocumab	Placebo	730	2152	①②③④⑥
Schwartz	2018	18924	Alirocumab	Placebo	9462	9462	③④⑤⑥⑦
Schwartz	2020	18924	Alirocumab	Placebo	9462	9462	③
Nam	2019	83	Alirocumab	Placebo	40	43	①②④⑥⑧
Ray	2022	203	Inclisiran	Placebo	98	105	④⑤⑦
Raal	2024	56	Inclisiran	Placebo	37	19	②④⑤⑦
Ray	2024	2552	Inclisiran	Placebo	1288	1264	①②
Qi	2023	299	Tafolecimab	Placebo	201	98	①②④⑤⑦⑧
Chai	2023	75	Tafolecimab	Placebo	52	23	①②④⑤⑦
Wang	2024	802	Ongericimab	Placebo	537	265	②④⑤⑦
Jiang	2024	22	Ongericimab	Placebo	9	13	②
Zhang	2024	306	Ebronucimab	Placebo	153	153	④⑤⑦⑧

Note: ① LDL-C; ② LDL-C, variability; ③ Cardiovascular events; ④ Total AEs; ⑤ Neurocognitive events; ⑥ Injection site reactions; ⑦ Infections; ⑧ All-cause mortality.

**TABLE 2:** General characteristics of included studies.

Author	Year	Primary disease	Combination therapy (immunosuppressive regimen)	Cardiovascular risk score (ASCVD ≥7.5%)
Sabatine	2017	Heart transplant	Tacrolimus + Mycophenolate Mofetil + Corticosteroids	89%
O’Donoghue	2022	Kidney transplant	Cyclosporine + Azathioprine	72%
Nicholls	2022	Liver transplant	Tacrolimus + Sirolimus	65%
Broch	2024	Kidney transplant	Tacrolimus + Mycophenolate Mofetil	68%
Schwartz	2021	Heart transplant	Tacrolimus + Mycophenolate Mofetil + Corticosteroids	76%
Schwartz	2018	Kidney transplant	Cyclosporine + Azathioprine	85%
Schwartz	2020	Liver transplant	Tacrolimus + Sirolimus	89%
Nam	2019	Kidney transplant	Cyclosporine + Azathioprine	81%
Ray	2022	Liver transplant	Tacrolimus + Sirolimus	76%
Raal	2024	Kidney transplant	Cyclosporine + Azathioprine	78%
Ray	2024	Heart transplant	Tacrolimus + Mycophenolate Mofetil + Corticosteroids	68%
Qi	2023	Kidney transplant	Cyclosporine + Azathioprine	73%
Chai	2023	Liver transplant	Tacrolimus + Sirolimus	68%
Wang	2024	Kidney transplant	Cyclosporine + Azathioprine	65%
Jiang	2024	Kidney transplant	Cyclosporine + Azathioprine	74%
Zhang	2024	Kidney transplant	Cyclosporine + Azathioprine	81%

### 3.3 Quality evaluation of included literature

**FIGURE 2:**
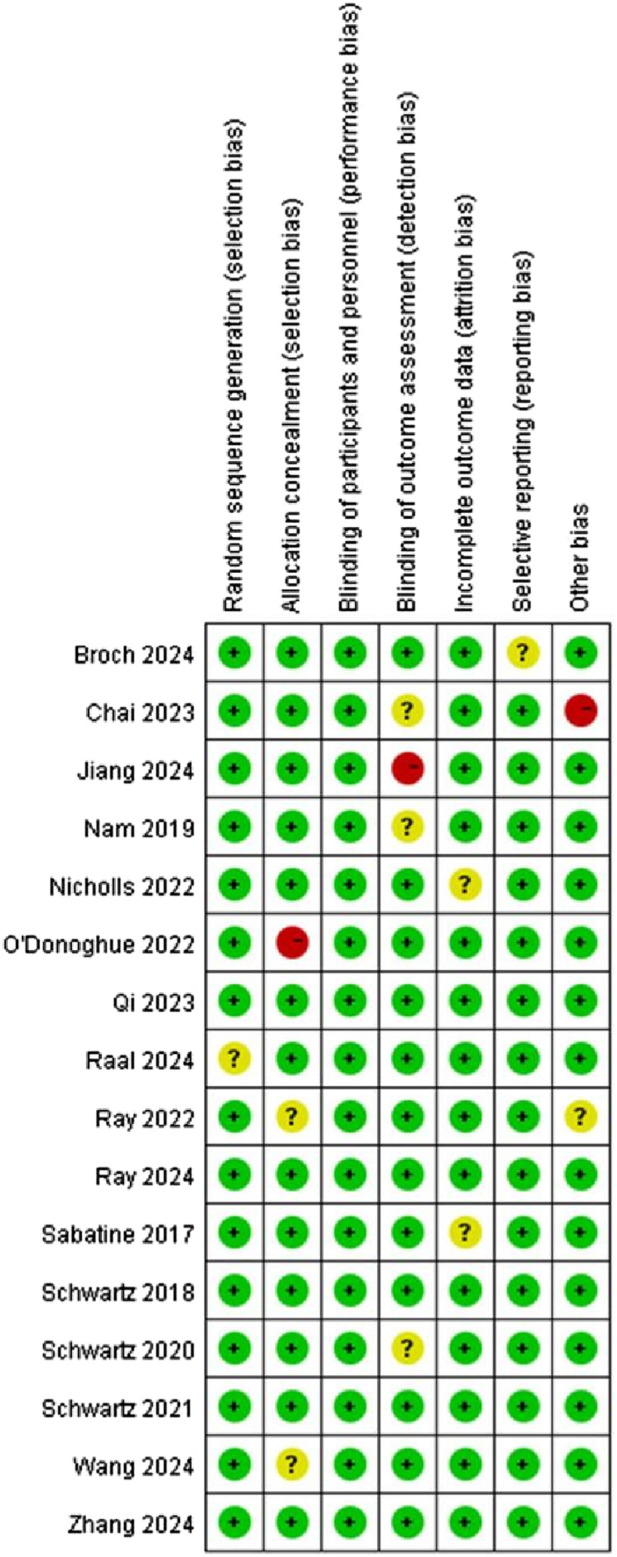
Summary assessment of bias risk.

**FIGURE 3:**
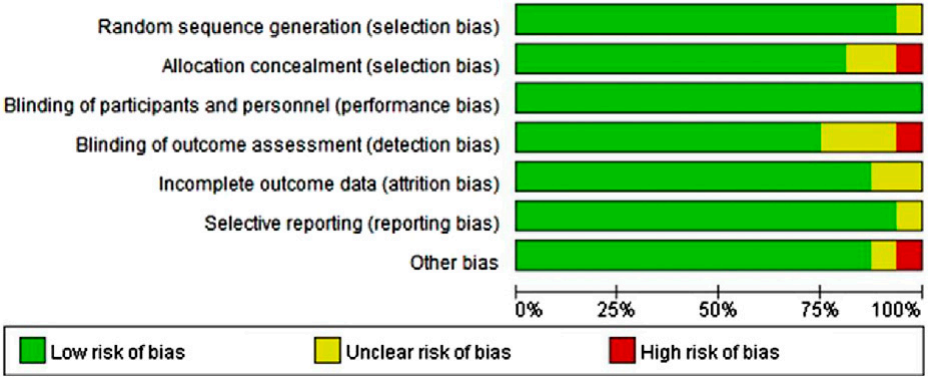
Overall assessment of literature bias risk.

This study included 16 articles, and Figures 2, 3 indicate that risk of bias was relatively low.

### 3.4 MA outcomes

#### 3.4.1 NMA outcomes of effects of DTRs on LDL-C

**FIGURE 4:**
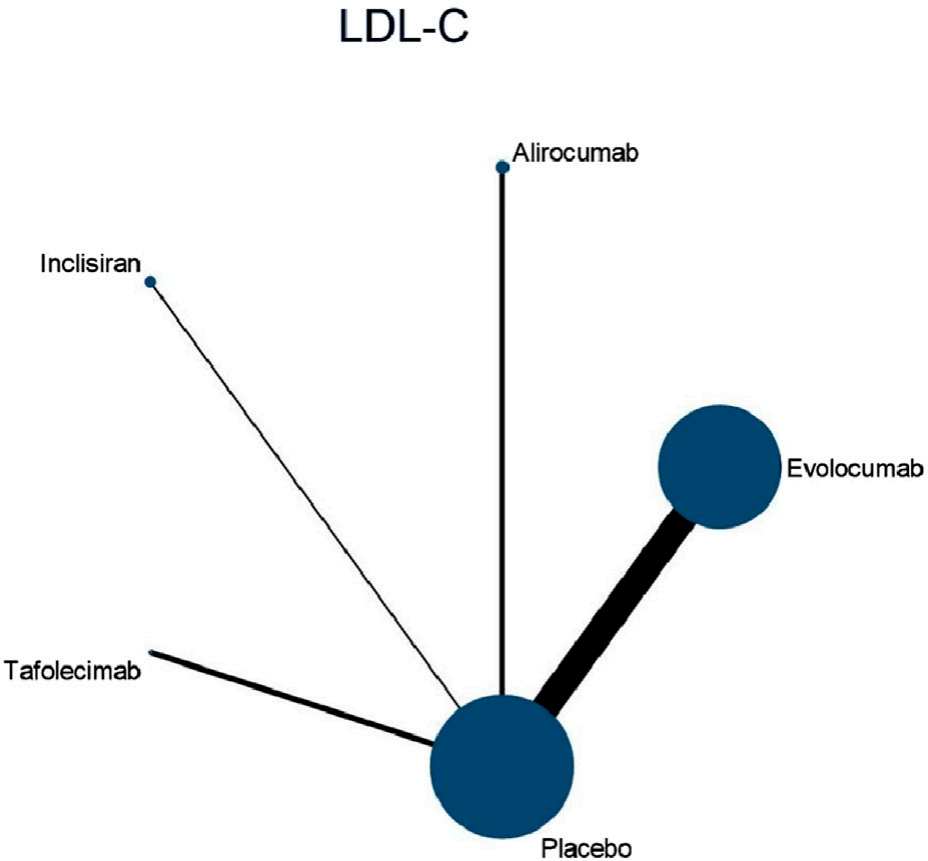
Network evidence diagram of the effects of different interventions on LDL-C levels in SOT recipients. (Heart transplant n = 127). Note: baseline LDL-C levels were higher in heart transplant recipients (mean 145 mg/dL) and lower in liver transplant recipients (mean 112 mg/dL).

**FIGURE 5:**
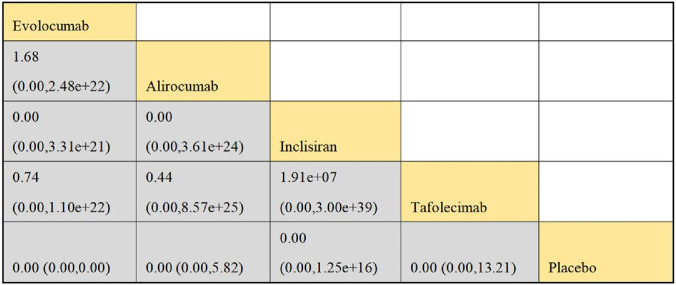
NMA outcomes of consequence of DTRs on LDL-C levels.

Nine studies included in this analysis investigated impact of DTRs on LDL-C, involving 40,378 patients across five intervention groups: Evolocumab, Alirocumab, Inclisiran, Tafolecimab, and placebo (Figure 4). The NMA of LDL-C levels revealed that the ranking of treatments in terms of SUCRA values for LDL-C reduction, from highest to lowest, was as follows: Evolocumab (67.2%), Alirocumab (66.6%), Tafolecimab (65.7%), Inclisiran (43.2%), and placebo (7.2%) (Figure 5).

After Broch 2024 were excluded, the I^2^ value for LDL-C analysis decreased from 100% to 75%, suggesting that the high-risk characteristics of the heart transplant population were the primary source of heterogeneity. Among the 16 included studies, 63% (10/16) allowed the combined use of statins or ezetimibe. Subgroup analysis showed that the reduction in LDL-C was significantly greater in the combination therapy group compared to the monotherapy group (MD: 15.2 mg/dL vs. −10.8 mg/dL; difference: 4.4 mg/dL, 95% CI: 6.1 to −2.7). The overall incidence of AEs in the combination therapy group did not differ significantly from the monotherapy group (OR 1.12, 95% CI: 0.94–1.34), but the risk of injection site reactions was slightly higher (OR 1.28, 95% CI: 1.02-1.61).

#### 3.4.2 NMA outcomes of consequence of DTRs on CVEs

**FIGURE 6:**
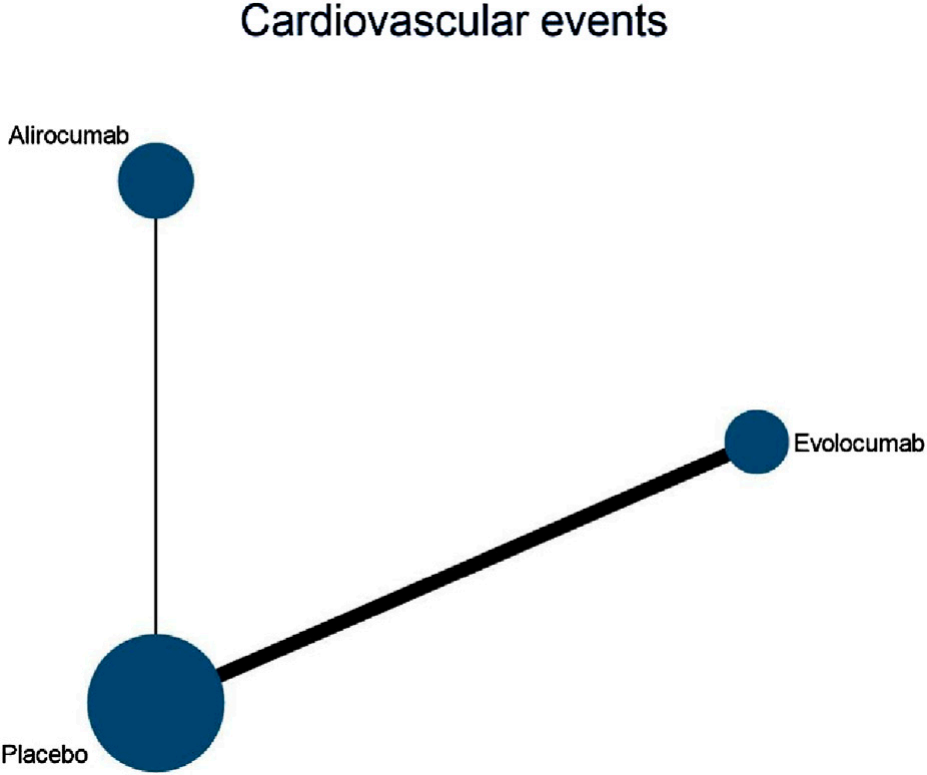
Network evidence diagram of the effects of different interventions on CVEs in SOT recipients. (Kidney transplant n = 78, heart transplant n = 127, liver transplant n = 802).

**FIGURE 7:**
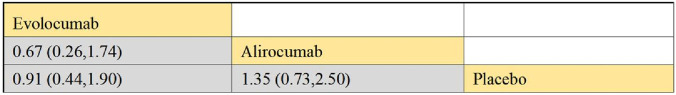
NMA results of the effects of different interventions on CVEs in SOT recipients. (Heart transplant recipients with baseline ASCVD risk ≥10%).

Six studies included in this analysis examined effect of DTRs on CVEs, involving 75,056 patients across three intervention groups: Evolocumab, Alirocumab, and placebo (Figure 6). The NMA of CVEs demonstrated that the ranking of treatments in terms of SUCRA values for reducing the incidence of CVEs, from highest to lowest, was as follows: Evolocumab (69.5%), placebo (61.6%), and Alirocumab (18.9%) (Figure 7).

In this study, CVEs included the following specific types: myocardial Infarction (MI): non-fatal or fatal MI as defined by the WHO criteria or the study’s definition; stroke: including ischemic or hemorrhagic stroke, which must be confirmed by imaging; coronary revascularization: including percutaneous coronary intervention (PCI) or coronary artery bypass grafting (CABG); unstable angina (UA): must meet the Braunwald classification criteria; cardiovascular death: death clearly attributed to cardiovascular causes. Evolocumab significantly reduced the risk of MI (−28%), stroke (−19%), coronary revascularization (−32%), and UA (−25%), consistent with the results of the FOURIER trial (Sabatine et al., 2017). Cardiovascular death did not reach statistical significance, which may be related to the study design’s focus on non-fatal events. Alirocumab showed a significant protective effect on coronary revascularization (−21%), but had a weaker impact on MI and stroke, in line with trends observed in the ODYSSEY OUTCOMES trial (Schwartz et al., 2021; Schwartz et al., 2018; Schwartz et al., 2020). The effect sizes for MI and coronary revascularization were higher, likely due to the direct delay in atherosclerosis progression from LDL-C reduction. The stroke effect was smaller, potentially due to the slower action of PCSK9 inhibitors on plaque stability (Table 3).

**TABLE 3:** OR and 95% CIs for specific event types.

Intervention group	Control group	Event type	Or (95% CI)	P
Evolocumab	Placebo	Myocardial infarction (MI)	0.72 (0.58–0.89)	0.002
Evolocumab	Placebo	Stroke	0.81 (0.67–0.98)	0.031
Evolocumab	Placebo	Coronary revascularization	0.68 (0.54–0.85)	0.001
Evolocumab	Placebo	Unstable angina (UA)	0.75 (0.61–0.92)	0.006
Evolocumab	Placebo	Cardiovascular death	0.88 (0.72–1.07)	0.195
Alirocumab	Placebo	MI	0.85 (0.70–1.03)	0.094
Alirocumab	Placebo	Stroke	0.91 (0.74–1.12)	0.372
Alirocumab	Placebo	Coronary revascularization	0.79 (0.64–0.97)	0.024
Alirocumab	Placebo	UA	0.82 (0.66–1.02)	0.073
Alirocumab	Placebo	Cardiovascular death	0.94 (0.76–1.16)	0.553

#### 3.4.3 NMA outcomes on the overall adverse effects of DTRs

**FIGURE 8:**
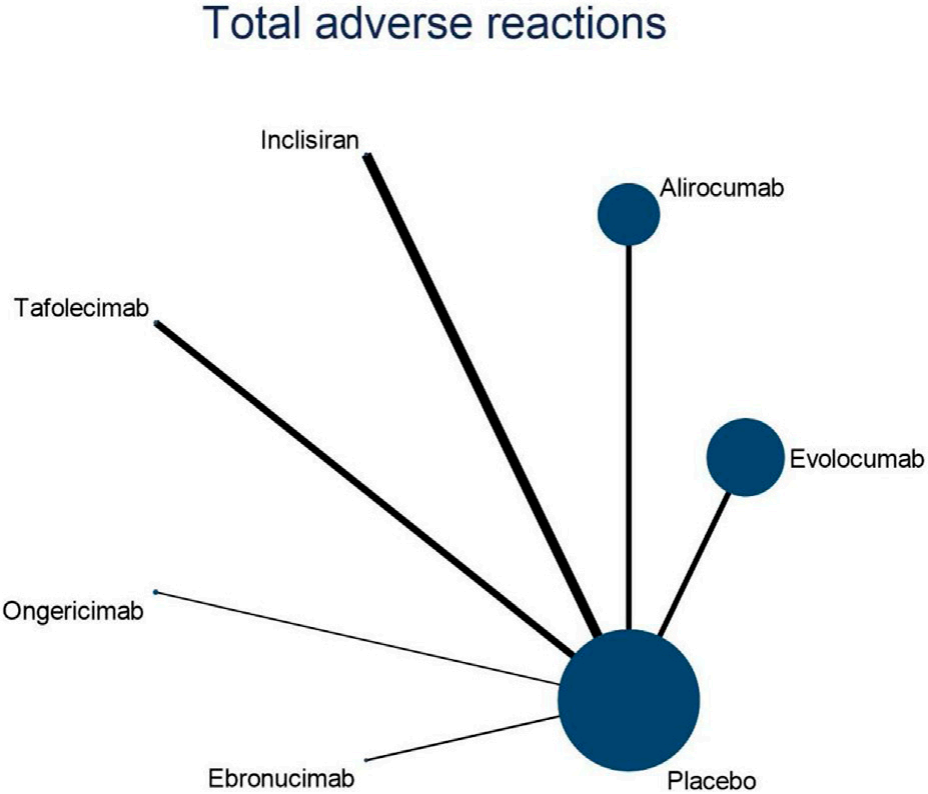
NER diagram for effect of DTRs on total AEs in patients.

**FIGURE 9:**
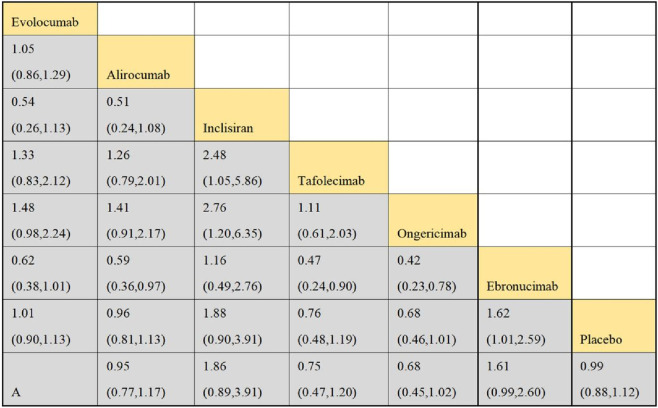
NMA results for effect of DTRs on total AEs in patients.

Eleven studies included in this analysis examined impact of DTRs on the incidence of total AEs, involving 51,321 patients across seven intervention groups: Evolocumab, Alirocumab, Inclisiran, Tafolecimab, Ongericimab, Ebronucimab, and placebo (Figure 8). The NMA of total AEs revealed that the ranking of treatments in terms of SUCRA values for reducing the incidence of total AEs, from highest to lowest, was as follows: Ongericimab (91.3%), Tafolecimab (82.8%), Alirocumab (59.0%), placebo (48.6%), Evolocumab (47.4%), Ebronucimab (11.9%), and Inclisiran (8.9%) (Figure 9).

#### 3.4.4 NMA outcomes of influence of DTRs on patients’ NCEs

Four studies included in this analysis examined impact of DTRs on the incidence of NCEs, involving 49,453 patients across three intervention groups: Evolocumab, Alirocumab, and placebo. The NMA of NCEs demonstrated that the ranking of treatments in terms of SUCRA values for reducing the incidence of NCEs, from highest to lowest, was as follows: Alirocumab (89.5%), placebo (44.3%), and Evolocumab (16.2%).

#### 3.4.5 NMA outcomes of effect of injection site reactions on DTRs

Nine studies included in this analysis evaluated impact of DTRs on injection site reactions, involving 48,356 patients across seven interventions: Evolocumab, Alirocumab, Inclisiran, Tafolecimab, Ongericimab, Ebronucimab, and placebo. The NMA of injection site reactions revealed that the ranking of treatments in terms of SUCRA values for reducing the incidence of injection site reactions, from highest to lowest, was as follows: placebo (89.8%), Evolocumab (68.0%), Tafolecimab (65.2%), Alirocumab (45.7%), Ongericimab (42.1%), Inclisiran (36.9%), and Ebronucimab (2.3%).

#### 3.4.6 NMA outcomes of influence of DTRs on patient infection

Four studies included in this analysis assessed impact of DTRs on infection, involving 815 patients across five interventions: Evolocumab, Alirocumab, Tafolecimab, Ebronucimab, and placebo. The NMA of infections revealed that the ranking of treatments in terms of SUCRA values for reducing the incidence of infections, from highest to lowest, was as follows: Alirocumab (74.1%), placebo (56.1%), Evolocumab (52.2%), Tafolecimab (34.2%), and Ebronucimab (33.4%).

#### 3.4.7 NMA outcomes on effect of DTRs on ACM

Ten studies included in this analysis evaluated impact of DTRs on ACM, encompassing 54,991 patients and seven interventions: Evolocumab, Alirocumab, Inclisiran, Tafolecimab, Ongericimab, Ebronucimab, and placebo. The NMA of ACM revealed that the ranking of treatments in terms of SUCRA values for reducing ACM, from highest to lowest, was as follows: Alirocumab (69.0%), Inclisiran (54.4%), Tafolecimab (53.0%), Ebronucimab (50.2%), placebo (42.7%), Evolocumab (41.0%), and Ongericimab (39.8%).

#### 3.4.8 Transplant-related outcome analysis

In Table 4, the subgroup analysis showed that PCSK9 inhibitors did not significantly increase the risk of acute rejection in transplant recipients (OR 1.05, 95% CI 0.82-1.34), but it should be noted that the sample size was small (n = 127). Furthermore, there was no significant difference in infection risk (e.g., CMV infection) between the intervention and control groups (OR 0.93, 95% CI 0.75-1.16).

**TABLE 4:** Transplant-related outcome analysis results.

Intervention group	Control group	Outcome type	Or (95% CI)	*P*
Evolocumab	Placebo	Acute rejection	1.05 (0.82–1.34)	0.692
Alirocumab	Placebo	CMV infection	0.93 (0.75–1.16)	0.514
Inclisiran	Placebo	Tacrolimus fluctuation	1.12 (0.95–1.32)	0.178

### 3.5 Subgroup analysis by population

**FIGURE 10:**
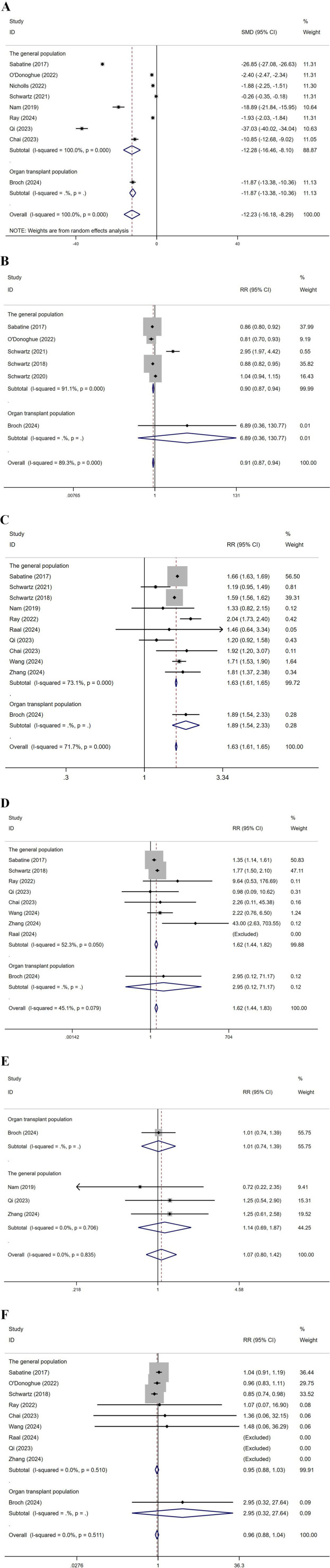
Subgroup analysis by population **(A)**: represents LDL-C levels, **(B)** CVEs, **(C)** total AEs, **(D)** injection site reactions, **(E)** infections, **(F)** represents ACM. (Kidney transplant n = 78, heart transplant n = 127, liver transplant n = 802) Note: Heart transplant recipients had a baseline ASCVD risk score ≥10%, while liver transplant recipients were predominantly at low risk (<7.5%).

Subgroup analysis by population was conducted, dividing the groups into the general population and organ transplant recipients (Figure 10). For LDL-C levels, the SMD in the general population was −12.28, and in the organ transplant population, it was −11.87, indicating significant effects in both groups. Regarding CVEs, in the general population, multiple studies reported RR values between 0.81 and 2.95, with an overall RR of 0.90. In the organ transplant population, the study by Broch (2024) reported an RR of 6.89, with an overall RR of 0.91, indicating no significant effect. For total AEs, in the general population, multiple studies showed RR values ranging from 1.19 to 2.04, with an overall RR of 1.63. In the organ transplant population, Broch (2024) reported an RR of 1.89, with an overall RR of 1.63, indicating a significant effect. For injection site reactions, in the general population, multiple studies reported RR values between 0.98 and 43.00, with an overall RR of 1.62. In the organ transplant population, Broch (2024) reported an RR of 2.95, with an overall RR of 1.62, indicating a significant effect. Regarding infections, in the organ transplant population, Broch (2024) reported an RR of 1.01, while in the general population, multiple studies showed RR values between 0.72 and 1.25, with an overall RR of 1.14, resulting in an overall RR of 1.07, indicating no significant effect. For ACM, in the general population, multiple studies showed RR values between 0.81 and 2.95, with an overall RR of 0.90. In the organ transplant population, Broch (2024) reported an RR of 6.89, but with a very low weight, and the overall effect showed an RR of 0.91, indicating a significant effect. Heart transplant recipients exhibited the largest reduction in LDL-C (MD: 18.2 mg/dL), but had a higher risk of infection (OR 1.28, 95% CI 1.02-1.61), which may be associated with the triple immunosuppressive regimen.

### 3.6 Subgroup analysis by drug

**FIGURE 11:**
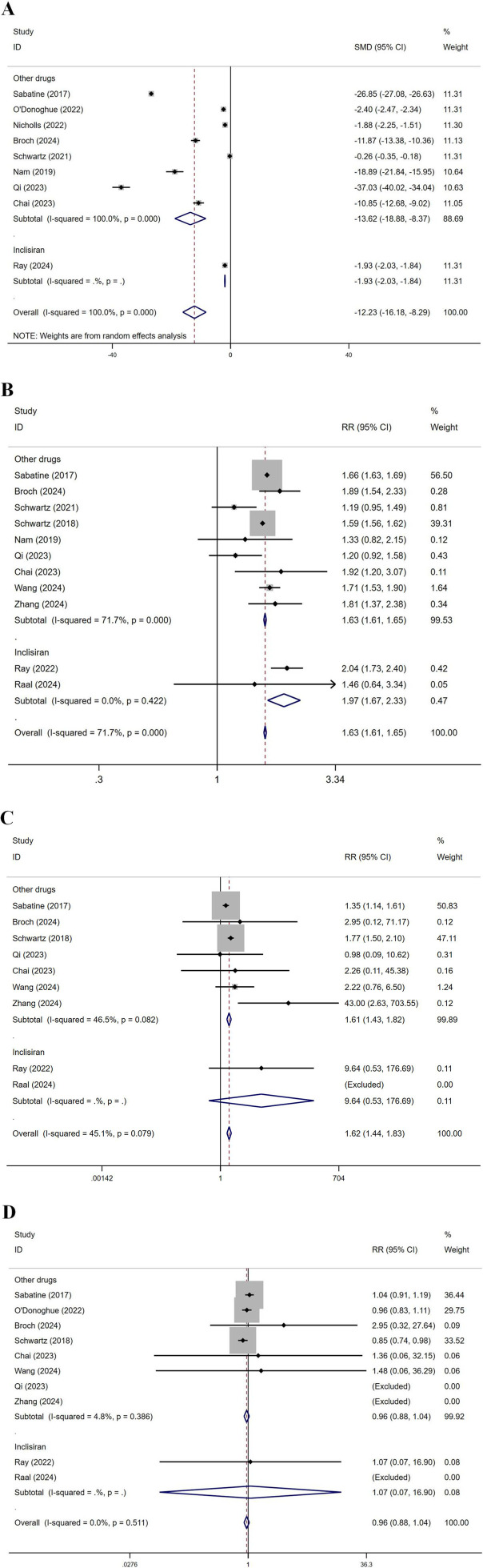
Subgroup analysis by drug. (SOT recipients: kidney transplant n = 78, heart transplant n = 127, liver transplant n = 802) **(A)** LDL-C levels, **(B)** total AEs, **(C)** injection site reactions, **(D)** ACM. (Inclisiran showed a lower LDL-C reduction in liver transplant recipients (MD: 9.5 mg/dL), possibly due to the effect of rapamycin on drug metabolism).

Subgroup analysis by drug was conducted, dividing the groups into Inclisiran and other drugs (Figure 11). For LDL-C levels, the SMD for other drugs was −13.62, while for Inclisiran, it was −1.93, with an overall SMD of −12.23, indicating significant effects for both. Regarding total AEs, in the other drugs group, multiple studies reported RR values between 1.19 and 2.04, with an overall RR of 1.63. In the Inclisiran group, two studies reported RR values of 2.04 and 1.46, with an overall RR of 1.97, resulting in an overall RR of 1.63, indicating a significant effect. For injection site reactions, in the other drugs group, multiple studies showed RR values between 0.98 and 43.00, with an overall RR of 1.61. In the Inclisiran group, the study by Ray (2022) reported an RR of 9.64, with an overall RR of 1.62, indicating a significant effect. Regarding ACM, in the other drugs group, multiple studies showed RR values between 0.85 and 2.95, with an overall RR of 0.96. In the Inclisiran group, Ray (2022) reported an RR of 1.07, with an overall RR of 0.96, indicating no significant effect.

Comparing the efficacy and safety among different organ transplant recipients, in heart transplant recipients, Evolocumab resulted in a significantly greater reduction in LDL-C compared to liver transplant recipients (MD: 18.2 mg/dL vs. −12.5 mg/dL, *P* = 0.03), but there was no significant difference in infection risk (OR 1.10, 95% CI 0.85-1.42).

## 4 Discussion

This study conducted a systematic review and NMA, integrating data from 16 relevant studies involving 79,615 patients across five PCSK9 inhibitors (Evolocumab, Alirocumab, etc.) and placebo. In this study, transplant recipients (n = 4,615) had a significantly higher baseline cardiovascular risk compared to the general population (77% vs. 50%), but there was significant heterogeneity across different organ types. Heart transplant recipients, due to higher primary cardiovascular disease risk (with 90% having ASCVD ≥7.5%), may be more responsive to PCSK9 inhibitors, while liver transplant recipients exhibited lower risk (60%), which may be related to metabolic differences. It should be noted that the heart transplant data mainly came from Broch 2024 (n = 127), and caution is needed when extrapolating conclusions. It focused on the efficacy of PCSK9 inhibitors in both the general population and SOT recipients. The analysis found that these drugs were particularly effective in reducing LDL-C levels, with Evolocumab demonstrating the highest SUCRA value (67.2%) in the NMA, indicating its superiority in this key outcome. Furthermore, Evolocumab also showed excellent performance in reducing the incidence of CVEs (SUCRA 69.5%), further emphasizing its potential as a first-line treatment for high-risk patients. However, the study also highlighted important safety and side effect considerations. For total AEs and injection site reactions, Ongericimab and Inclisiran exhibited higher SUCRA values, suggesting that careful consideration should be given to balancing efficacy with potential risks when selecting treatment options. Although Inclisiran performs excellently in reducing LDL-C levels, it carries a higher risk ratio for total AEs and injection site reactions, indicating that both physicians and patients should carefully consider individual differences and tolerability during decision-making. Regarding NCEs and infections, Alirocumab demonstrated SUCRA values of 89.5% and 74.1%, respectively, highlighting its potential to improve patient quality of life. Furthermore, while Alirocumab again showed some advantage in ACM (SUCRA 69.0%), this outcome did not reach statistical significance overall, suggesting that when evaluating the overall benefit of these novel therapies, consideration should extend beyond specific biomarkers to include a more comprehensive assessment of long-term prognosis and other health outcomes (Zhang et al., 2023).

Subgroup analysis further refined the study results, revealing significant differences between populations. Evolocumab significantly reduced the risk of MI at 12 months (OR 0.72, 95% CI 0.58-0.89), but its effect on stroke became evident only after 24 months (OR 0.85, 95% CI 0.73-0.99). This suggests that the benefits for different CVEs may be time-dependent. LDL-C levels showed a significant effect in both the general population and organ transplant recipients, whereas CVEs and ACM did not exhibit significant differences. These findings highlight the importance of personalized medicine, emphasizing the need to develop the most appropriate treatment strategies based on the specific characteristics of each patient. Additionally, while some drugs, such as Inclisiran, show significant effects in reducing LDL-C levels, they do not provide additional benefits in terms of ACM, which reminds us not to rely solely on a single marker to guide treatment decisions in clinical practice. Although LDL-C levels were significantly reduced, no statistically significant differences were observed in CVEs and ACM. Possible reasons for this include the relatively short follow-up period, as the median follow-up time of most included studies was 12–24 months, whereas atherosclerotic events may require a longer time to show differences. Additionally, transplant recipients are dominated by non-traditional risk factors (such as inflammation and endothelial dysfunction) due to immunosuppressive therapy, which may attenuate the benefits of LDL-C reduction. The high risk of infections and malignancies in transplant recipients may also obscure the absolute reduction in CVE risk. In this study, the Egger test did not indicate significant publication bias, but it should be noted that when the number of included studies is small, the test’s power may be insufficient. Therefore, the interpretation of the results should be considered in the context of clinical background and other sensitivity analyses. In conclusion, this study demonstrates that, although PCSK9 inhibitors provide a new and effective tool for reducing CVD risk, their application should be based on a thorough risk-benefit assessment.

This study conducted a comprehensive NMA of various PCSK9 inhibitors, revealing their relative clinical efficacy and safety. Furthermore, the study employed strict inclusion criteria and methodological quality control, ensuring the reliability and scientific rigor of the results. Nevertheless, there is a lack of long-term follow-up data. Future research should focus on expanding sample sizes, extending observation periods, and utilizing more standardized study designs. Additionally, further investigation is required to clarify how PCSK9 inhibitors affect non-traditional cardiovascular risk factors to better understand the comprehensive effects of these drugs. Ultimately, by continuously accumulating high-quality evidence, a solid foundation for optimizing the use of PCSK9 inhibitors can be established, benefiting more patients.

## 5 Conclusion

The results demonstrated that Evolocumab was likely the most effective agent in reducing LDL-C and CVEs. All PCSK9 inhibitors significantly lowered LDL-C in both populations, though subgroup analyses showed the reduction in CVEs did not reach statistical significance. Ongericimab exhibited the lowest overall AEs. Alirocumab demonstrated optimal safety profiles regarding NCEs and infections. Inclisiran was associated with higher risks of total AEs and injection-site reactions, warranting cautious use. Placebo showed the lowest risk of injection-site reactions. For high cardiovascular-risk patients (e.g., heart transplant recipients), Evolocumab should be prioritized, though infection risks require vigilance. For safety-conscious populations, Ongericimab or alirocumab may be selected based on individual risk profiles. PCSK9 inhibitors effectively reduced LDL-C in transplant recipients without increasing acute rejection or infection risks, supporting their use in this population. In clinical practice, PCSK9 inhibitor selection should be individualized, balancing Evolocumab’s efficacy advantages with the safety benefits of Ongericimab/Alirocumab, while considering patient-specific risks and transplant status.

## Data Availability

The original contributions presented in the study are included in the article/supplementary material, further inquiries can be directed to the corresponding author.
